# Managing and Analyzing Next-Generation Sequence Data

**DOI:** 10.1371/journal.pcbi.1000369

**Published:** 2009-06-26

**Authors:** Brent G. Richter, David P. Sexton

**Affiliations:** 1 Enterprise Research IS and Informatics, Brigham and Women's Hospital, Massachusetts General Hospital, and Partners Healthcare, Boston, Massachusetts, United States of America; 2 Center for Human Genetics Research, Computational Genomics Core, Vanderbilt University, Nashville, Tennessee, United States of America; University of California San Diego, United States of America

## Introduction

Centralized Bioinformatics Core Facilities provide shared resources for the computational and IT requirements of the investigators in their department or institution. As such, they must be able to effectively react to new types of experimental technology. Recently faced with an unprecedented flood of data generated by the next generation of DNA sequencers, these groups found it necessary to respond quickly and efficiently to the informatics and infrastructure demands. Centralized Facilities newly facing this challenge need to anticipate time and design considerations of necessary components, including infrastructure upgrades, staffing, and tools for data analyses and management.

The evolution of the sequencing instrumentation is far from static. Sequence throughput from this new generation of instruments continues to increase exponentially at the same time that the cost of sequencing a genome continues to fall. These realities make the technology accessible to greater numbers of investigators while leading them to a greater usage of sequencing for a variety of experimental techniques, including variation discovery, whole transcriptome analysis, and DNA–protein interaction analysis. This places unique challenges upon the Bioinformatics Core Facility, whose mission could vary from the support of a single department or sequencing core to a Facility that supports many disparate and independent groups that run their own sequencers but rely on the Central Facility to host the informatics, research cyberinfrastructures, or both. It is worth noting that the initial investment in the instrument is accompanied by an almost equal investment in upgrading the informatics infrastructure of the institution, hiring staff to analyze the data produced by the instrument, and storing the data for future use. Many investigators do not realize that these extensive investments are necessary prior to purchasing the new technology. This is why it is advantageous to have a centralized Bioinformatics Core to put in place platforms that acquire, store, and analyze the very large datasets created by these instruments. A Bioinformatics Core, already familiar with data of this type and complexity, dedicated to investigators, and jointly working with IT personnel, can span multiple domains rather effortlessly.

The large sequencing centers (e.g., Sanger, Broad Institute, and Washington University) have automated processes and architectures not generally replicable in medium and small sequencing groups. However, as these smaller groups obtain next-generation technology they can nevertheless learn lessons from the larger centers. Through collaboration and sharing best practices, small and medium-sized groups can prepare for the arrival of the technology and develop methods to manage and analyze the data. The Bioinfo-Core Special Interest Group [1], affiliated with the International Society for Computational Biology, has been actively collaborating to formulate best practices to assist small and medium-sized Cores in setting up platforms for next-generation sequencing. Here, we provide a Perspective for such a Core Facility in accomplishing this task, using collective experiences from Facilities that have solved many of these issues.

## Background

Several new sequencing methodologies have been developed, most of which are loosely based on fixing DNA sequences to glass beads or slides, amplification and tagging of the bases with compounds for visualization, image capture, and subsequent image analysis to derive base calls. Some of the techniques and manufacturers include sequencing by synthesis as used by the Solexa Genome Analyzer II (GAII) by Illumina, sequencing by ligation as used by the ABI SOLiD sequencer and by the polony sequencing technique developed by the Church Lab at Harvard Medical School, sequencing by hybridization as used by Affymetrix, and single molecule sequencing as used by Helicos, VisiGen, and Pacific Biosciences. As of this writing, the preponderance of data has come from the GAII, which currently has the largest market penetration and is clearly the most established next-generation sequencing technology among the members of our Special Interest Group.

The uniqueness of these data stems from the number of files created and the size of those files generated during a sequencing run. For the GAII system, approximately 115,200 Tiff formatted files are produced per run, each at about 8 megabytes (MB) in size. This is approximately 1 terabyte (TB) of data, which must be moved from the capture workstation to the analysis resource. Other systems have similar data and image yields [2],[3]. A decision must be made about archiving these “raw” data for future analysis or discarding them in favor of resequencing. A mere 10–20 sequencing runs could overwhelm any storage and archiving system available to individual investigators. Analysis of the image files is accomplished by Illumina-provided software or by any number of third-party applications. Since the instrument is typically run for 36 cycles, sequences of about 36 bases are produced, resulting in what are called short read sequences. Sequence of this length creates major impediments to assembly of complex genomes without the use of a reference. Currently, de novo assemblies are restricted to prokaryotic and BAC genomes.

Even after image processing, basecalling, and assembly, there will be approximately 300 GB of uncompressed primary data that must be stored either in flat files or in a database. Then, using public databases and tools, biological significance can be assigned to the sequence. Many of the current algorithms and software programs are unable to handle the number and size of the sequence reads that must therefore be modified for use. Currently, software to reliably visualize the sequence data and its assemblies is evolving. Additionally, the long-term storage of primary and derived data may be difficult for the investigator, necessitating centralized solutions.

Solutions to these issues can be accomplished with a small, dedicated group within organizations that are familiar with data of this type and complexity. Within each area, we will describe specific challenges, along with some possible solutions we have experienced ourselves and from the experience of other institutions. These may not be the only solutions or architectures, and there are certainly many and varied sources of information on these topics as the target requirements continue to move, but this Perspective can serve as a starting point for a set of best practices derived from Facilities that have already solved many of these issues.

## Getting Started with the Next-Generation Manufacturers

The current instrument manufacturers—Illumina, Roche, and Applied Biosystems—all provide a foundation workflow for running their systems. Instruments typically ship with modest compute and IT resources providing the ability to support a single run of the machine. A small cluster, server, or workstation directly attached to the instrument provides data capture along with the principal data analysis pipelines necessary to process the raw data acquired into base calls and sequence alignments from the run itself. Lately, manufacturers are also providing additional analysis modules, complete with technical support, to help streamline the Primary Analysis pipeline. In most buying considerations, the purchase of these additional modules provides an immense overall cost savings to the small and medium-sized Core. In the case of the Solexa GAII, this translates into a small incremental investment for the IPAR module, which significantly shortens the overall run time as well as providing diagnostics of the image analysis pipeline through bundled technical support.

As researchers and Core Facilities obtain more sequencers and are required to capture and store more than a single run at a time, they will need to grow quickly into larger compute and storage infrastructures capable of supporting these additional needs as well as information management systems to manage not only the workflow and derived information but also the data itself. Although the next-generation instruments are becoming widespread throughout academic institutions and medical centers, they are still an emerging technology. Solexa sequencing, for example, has been available to the small-to-medium-sized Cores since the summer of 2007. To date, technologists, IT groups, and informaticians have had a relatively short period of time in which to develop processes, best practices, and additional, more rigorous QC/QA and LIMS environments specific to their environments. As these technologies and algorithms emerge into academic, open-source, and vendor-supported offerings, Core Facilities will evaluate them against existing practices using previous datasets.

Additionally, the manufacturers themselves are rapidly developing their platforms with frequent improvements to their technology and informatics solutions. This may require re-analysis using the technology for new insights or at minimum a QA of the new revisions against older software versions using previously acquired data. This will continue to be the case as the scientific community demands longer individual read sequences and the manufacturers respond with changes and updates to optics, software, and chemistry, placing larger demands on institutions' IT requirements [4].

Because of the necessarily tight integration with IT, those Bioinformatics Facilities that don't already maintain their own research IT infrastructures, including hardware and systems administration resources, will need to lean heavily outside themselves, either on centralized institutional services, specialized computer consulting groups, or both. A final consideration for startup is accessibility to the sequencing facilities by these additional personnel. Troubleshooting technical issues during setup, configuration, and operation of these instruments will be necessary to assist lab operations.

## Computational Considerations

Moving beyond the initial installation, the transcendent requirement for a Facility's cyberinfrastructure is flexibility. Given the rapidly changing environment described, the manufacturer may or may not initially provide a modest computational environment, slating this environment for a subsequent release or update of the instrument. Consequently, the computational resources will need to fill technical gaps now and be able to scale for future demand.

The Solexa analysis pipeline, consisting of image analysis, base calling, and initial alignment against a reference sequence, initially was shipped without a computational platform upon which to run it. Most Bioinformatics Facilities either bought a large multiprocessor server or a small cluster into which the pipeline was configured. The current generation of the Solexa system can be shipped with an optional IPAR (Integrated Primary Analysis and Reporting) system consisting of a preconfigured 4-core server with 3 TB of usable storage. Intended for real-time use as a run completes, the system currently performs the image analysis step with the additional steps performed elsewhere. Illumina additionally provides a pipeline server, 16-cores, and a 9 TB disk Array that hosts the additional components of the pipeline.

This configuration provides a computational starting point. It usually becomes necessary either to scale up the vendor-provided system or to perform offline, primary analysis. Troubleshooting the analysis pipeline, manipulating configuration or parameter files, QAing revisions to the pipeline, or evaluating different algorithms requires a separate compute environment so that resources attached to the instrument can be used for the continued sequencing runs.

Two examples of initial configurations that have been successful are based on blades or discrete servers, respectively, and, through hardware miniaturization, products consisting of either solution can be initially hosted in a laboratory environment. The first is based on a small 8-node blade cluster (a node for each channel of the GAII) that can scale out as the number of instruments increase within the environment. This redundancy can also serve as backup if the IPAR module is down. In more modest environments, two identically configured generic 8-core servers with 6 TB storage and 32 GB RAM have been utilized to host the computational and storage needs. Additionally, these could serve for scale out through clustering at a later point.

## Data Dynamics

Storage and management of this data is arguably the largest issue with which a Facility will struggle. The principal needs are threefold: scalable, highly dense, and inexpensive disk systems for massive online growth; high-performance disk systems that place the data near to the pipeline algorithms; and archival storage for the data that are required to be kept by the institution. The difficult challenge in building such systems is the dichotomy between being able to handle a very large number of files that are accessed infrequently after primary analysis—with the expectation of online accessibility when the demand arises—and the need to provide high-performance access during analysis. One solution does not fit all requirements. Tradeoffs between inexpensive, highly dense storage using commodity disks and higher cost, highly performing NAS or SAN systems are dependent upon budget for many facilities. The balance between these is determined by reliability, performance, and budget. Prioritizing dollars can be difficult, but scalable systems that can grow along with storage requirements are most cost-effective for density along with purchasing a small yet high-performance NAS or SAN for transient analytical workloads. Many compromises can be made in the architectures, but we detail all components for completeness. Finally, centralized cyberinfrastructures make economical sense when scaling beyond two instruments and the manufacturers' initial offerings. This is especially true when the Bioinformatics Facility is required to support several disparate scientific groups whose requirements are guaranteed to change as these instruments continue to evolve and new experimental uses for the systems are developed.

High-density storage systems allowing for ad hoc growth into the petabyte range exist. These modular yet integrated storage environments provide several hundred terabytes of inexpensive disk provisioned in modules or blocks, aggregated together through software. Based on inexpensive SATA or SAS disks, both commercial and open solutions are available. Both are based on defined storage modules that can be stacked together over time as storage demands increase. Commercial solutions are usually integrated with software that provides aggregation of disks across the modules into one or a few very large file system namespaces. The open solutions, such as Lustre or GlusterFS, provide the aggregation layer, with commodity storage servers providing the storage blocks. There are additionally commodity solutions available based on independent storage servers integrated with open software such as Lustre or GlusterFS. This storage system will capture data while that data is being processed through various analysis pipelines. Because the data may only need to exist in this environment during analysis phases, the data itself can be considered transient and temporary within this system. Initially for budget considerations, a small storage footprint could be purchased, enough to house three data runs per instrument (6 TB).

An important consideration for the online, massive storage environment is the length of time necessary for the Facility to retain data. A group that understands the institutional requirements of the various sets of data (images, intensities, base pairs, and alignments) can develop reasonable data retention policies. Images, for example, may be retained long enough for primary analysis and QC to complete, then deleted—they may never touch a central file server. In some cases, the cost of the DNA sample and isolation is insignificant to the cost of DNA sequencing such that it will be cheaper to rerun than to store. However, in a clinical setting the DNA sample itself may be unique and therefore priceless, necessitating the need to store much of the upstream data.

Other Facilities that serve larger and more diverse communities, operating under defined service levels, may set policies to retain images for a specific period of time—three months, for example. In these situations, it will be necessary to initially determine the amount of storage required for three months of images and accompanying, derived data. In an average three-instrument environment operating during research business hours, this policy would require approximately 65 TB of usable storage, 200 TB if running the instruments at maximum throughput with maximum data capture, probably an unrealistic scenario in practical usage. Adding post-image analysis data, this figure can climb modestly to 75 TB. If images are removed immediately after processing, these figures drop to 10 TB.

Archival needs depend entirely upon the data-retention requirements. It is reasonable to retain all derived data within a terabyte-scale file system. However, due to regulatory or sample cost, it might be necessary to maintain a larger petabyte-scale tape or high-density disk storage system for diagnostics or personalized medicine, for example.

In addition to storage, there are other significant technical considerations that need to be resolved, primarily in networking and routine management of very large file systems. The systems and storage need to be simultaneously connected to several different networks. These range from institutional LAN connections to private networks. The centralized high-density storage will need to accept data arriving to it via LAN-connected instruments. Additionally, it may need to be connected to private networks serving computational or general-purpose cloud computing environments for further analysis or dissemination of derived information, respectively. A 1 GB network is essential within this environment, with 10 GB networks becoming more prevalent as the demands increase (and cost decreases). Raw network bandwidth, however, can be a small determinant to overall performance. Many technical decisions will be required during design and growth; and with the network interface typically outside the domain of a Core Facility, collaboration and careful negotiation, in balance with security, may play a role.

Finally, recovering a very large file system poses some very interesting challenges that certain IT vendors are addressing. A file system check on several hundred terabytes may require weeks to perform.

## Software and Post-Analysis

This area is by far the most rapidly evolving and most critical to providing useful information from these instruments as well as managing lab processes and data management of the raw and derived data. Software and informatics pipelines for principal analysis and visualization are in rapid development from both commercial sources and from the academic community.

The early adopters of these technologies, the very large sequencing centers, and later the medium-sized Core Facilities, understand the challenges they face with instruments of this type. The immediate challenge comes with a lack of adequate vendor-supported software and Laboratory Information Management Systems (LIMS). Early-stage Facilities rely heavily on custom-developed LIMS and informatics platforms. Given the tremendous cost and complexity of developing commercial-class LIMS modules with adequate flexibility built into the system for integration to internal business processes across many organizations, most instrument manufacturers do not provide such systems. However, some do provide an API or Web service interface to their software.

To the small and mid-sized Facilities, however, this is a very large gap in support, but that gap is shrinking. There exists a plethora of workflow applications, algorithms, and analysis pipelines in the public domain as well as commercial products coming to market. We will not attempt to summarize all the available software offerings, but, through Internet resources, the Bioinfo-Core list, and other blogs, and the recent flurry of new publications within the scientific and informatics literature, more than enough information is available [3].

For the purposes of this Perspective, the critical area for a Core will be in the integration of the principal analysis pipelines with data management and information delivery systems within organizations. Facilities are tasked with delivering data to research projects for additional analysis. The format of the data delivered range from short sequence reads to sequence that has been aligned to a reference. As the data volumes increase, there will be a greater demand on Facilities to fundamentally understand the uses of these machines in research so as to deliver the data in more useful ways other than raw sequence. Assignment of biological function and annotation of the sequence with features of interest will still be critical tasks. The methods to perform these tasks are still in the initial phases of development, with a few tools showing early promise [5].

As the cost of sequencing continues to decline, these technologies will translate into clinical settings and the area of personalized medicine, where the integration of this information with enterprise and personalized medical records, sample repositories, and knowledge management systems within medical institutions will be an absolute requirement to healthcare delivery and diagnostics. Other research environments are likely to encounter similar challenges soon.

## Staffing Requirements

There are many challenges in integrating next-generation sequencing instruments into the information technology infrastructure. Along with technology considerations, it is additionally critical to have a well-trained cadre of bioinformatics specialists operating within the Core, accessible to the entire institution in order to best serve the needs of those using this new technology. If the Core Facility has expertise in IT or can leverage other institutional resources for architecting and managing the IT systems described, then much of the operational work will involve bioinformatics analysis and systematizing the infrastructure. Specifically, these involve optimizing data analysis pipelines in the parallel computing environment, automating bulk transfers of large volumes of data, filtering data and assigning biological significance, interacting with investigators to understand the purpose of sequencing projects, and the ability to suggest analysis methods to investigators.

The skills necessary within the Facility include the following.

1. An intimate knowledge of UNIX-based operating systems.
2. Understanding of a scripting language such as Perl.
3. An understanding of parallel computing environments for UNIX clusters.
4. Knowledge of network-based data storage.
5. General knowledge of biology and genome sciences.
6. Ability to derive data analysis and software requirements from investigators who do not have a sophisticated understanding of information technology.
7. Ability to develop software encapsulating new analysis methods.
8. Understanding of relational databases and database architecture.
9. Ability to seek out and test novel bioinformatics software and analysis routines.

Finding a single staff member with all these skills would be extremely difficult, but finding members who have a subset of these skills and overlapping them in a team will be a more reasonable prospect. Individuals with these skill sets are rare and demand for their services is high, so compensation for such individuals is above that of laboratory technicians and bioinformaticians who have not operated in a high performance–computing environment. As such, a significant portion of the total cost of ownership for a next-generation sequencing operation will comprise staff member salaries.
